# Demand for weekend outpatient chemotherapy among patients with cancer in Japan

**DOI:** 10.1007/s00520-020-05575-x

**Published:** 2020-07-04

**Authors:** Hideki Katayama, Masahiro Tabata, Toshio Kubo, Katsuyuki Kiura, Junji Matsuoka, Yoshinobu Maeda

**Affiliations:** 1 Department of Palliative and Supportive Care, Okayama University Hospital, Okayama, Japan; 2 Clinical Cancer Center, Okayama University Hospital, Okayama, Japan; 3 Department of Allergy and Respiratory Medicine, Okayama University Hospital, Okayama, Japan; 4 Department of Hematology and Oncology, Okayama University Hospital, Okayama, Japan

**Keywords:** Weekend chemotherapy, Outpatient, Social burden, Cancer patient

## Abstract

**Background:**

Advanced cancer therapeutics have improved patient survival, leading to an increase in the number of patients who require long-term outpatient chemotherapy. However, the available schedule options for chemotherapy are generally limited to traditional business hours.

**Method:**

In 2017, we surveyed 721 patients with cancer in Okayama, Japan, regarding their preferences for evening and weekend (Friday evening, Saturday, and Sunday) chemotherapy appointments.

**Results:**

A preference for evening and weekend appointment options was indicated by 37% of the respondents. Patients who requested weekend chemotherapy were younger, female, with no spouse or partner, living alone, employed, and currently receiving treatment. Among these factors, age and employment status were significantly associated with a preference for weekend chemotherapy, according to multivariate analysis.

**Conclusion:**

Our findings reveal a demand for evening and weekend outpatient chemotherapy, especially among young, employed patients.

## Introduction

Advanced cancer therapeutics have improved patient survival but have also led to an increase in the number of patients who require long-term outpatient chemotherapy [1–3]. Treatment of patients with cancer in an outpatient setting is important for reducing the social burden of therapy and for maintaining quality of life (QoL) among these patients, as it allows them to integrate treatment into daily life [4–7]. However, outpatient chemotherapy often involves an extended duration of treatment, frequent hospital visits, long examinations before treatment, and (in some instances) prolonged infusion of anticancer drugs [8]. Consequently, outpatient treatment may affect patients’ daily life [9, 10]. The burdens associated with numerous extended-duration chemotherapy appointments may be partially mitigated by accommodating the patients’ lifestyles, such as by offering evening or weekend outpatient chemotherapy. Most Japanese hospitals, especially cancer treatment hospitals, offer outpatient chemotherapy only during weekday business hours. While a few hospitals currently offer weekend outpatient chemotherapy, the patient demand for this service has not been formally evaluated. The aims of this study were (i) to assess whether there is a substantial demand for evening or weekend outpatient chemotherapy and (ii) to identify the sociodemographic and clinical factors of patients with a preference for evening or weekend outpatient chemotherapy.

## Methods

Okayama Prefecture is a prefecture in Japan with a total population of approximately 1.9 million (approximately 1.2 million reside in two major cities, Okayama and Kurashiki). In a 2017 survey, 29.6% of the population was ≥ 65 years of age. Approximately 5600 people in the prefecture die from cancer each year. Our study was based on the results of a questionnaire sent to all designated cancer hospitals in Okayama Prefecture.

From August to September 2017, we conducted an anonymous, cross-sectional survey of the patients at 13 designated cancer hospitals (listed in Acknowledgements) in Okayama Prefecture, by means of a questionnaire distributed to outpatients ≥ 20 years of age who were currently undergoing treatment for cancer. Survey items included basic demographic information (i.e., age and sex), social background (i.e., marital status, cohabitation status, residence, employment, and annual personal income), and cancer characteristics (i.e., cancer type, current treatment status, and duration of treatment). Patients were also queried regarding their desire for evening and weekend (i.e., Friday evening, Saturday, or Sunday) outpatient chemotherapy. Personal income was stratified into annual income of < $20,000, $20,000–$39,999, and ≥ $40,000, according to the currency conversion rate at the end of the survey (US $1 = ¥112.47, 30 September 2017). The age at diagnosis and duration of treatment for patients with multiple cancers were defined as the age at diagnosis of the first cancer and the total duration of all cancer treatments, respectively. After the questionnaire had been completed by outpatients at each hospital, it was returned by mail to our hospital.

The interest of the survey respondents in weekend outpatient chemotherapy was assessed, and the relationships between patients’ sociodemographic and clinical factors were then analyzed. Statistical analysis was conducted using SPSS Statistics, version 25.0 (IBM Corp., Armonk, NY, USA). The chi-square test was used for comparison, with differences considered significant at *p* < 0.05. Multivariate logistic regression analysis was performed using factors identified as significant in univariate analysis. The study protocol was approved by the Institutional Review Board of each participating hospital.

## Results

The questionnaire was distributed to a total of 1500 patients; of these, 721 responded (48.1%). A preference for weekend chemotherapy was indicated by 36.5% of the respondents; the most common request was a Saturday appointment option (Table 1).

**Table 1:** Desire for Friday evening and weekend chemotherapy

		*N*		%
No request		458		(63.5)
Request		263		(36.5)
	Friday night*	117		
	Saturday*	184		
	Sunday*	145		

*multiple answers

Sociodemographic and clinical characteristics of the patients and the relationship of each factor with the desire for weekend chemotherapy are summarized in Tables 2, 3, and 4. A large number of questionnaire respondents were women, although men are generally more likely to develop cancer. The high number of women among the respondents was presumably because of the high response rates among patients with breast cancer (25%) and gynecological cancers (7%). Because these cancer types often affect younger people than other cancers [11, 12], the women who participated in the study were 8 years younger than the men who participated in the study. The average ages of cancer onset were 55 years for women and 63 years for men (Table 3). This clinical characteristic may have led to bias in the sociodemographic characteristics of patients with cancer in this study [12].

**Table 2:** Patient sociodemographic characteristics and results of univariate analysis

		Total		Request			
Characteristics		*N*	(%)	No	Yes	OR	*p* value
Age group at time of study							
	Less than 40 years	19	(2.6)	8	11		
	40–59 years	254	(35.2)	109	145		
	60–79 years	400	(55.5)	300	100		
	More than 80 years	43	(6)	37	6	82.36	< 0.001
	Missing	5	(0.7)				
Gender							
	Male	309	(42.9)	219	90		
	Female	410	(56.9)	238	172	12.51	< 0.001
	Missing	2	(0.3)				
Residence status							
	Living in Okayama	647	(89.7)	413	234		
	Moved to Okayama	25	(3.5)	12	13		
	Live in another prefecture	44	(6.1)	29	15	2.72	0.256
	Missing	5	(0.7)				
Marital status							
	Spouse/partner	544	(75.5)	361	183		
	No	175	(24.3)	97	78	6.84	0.009
	Missing	2	(0.3)				
Living situation							
	Live alone	53	(7.4)	32	21		
	One housemate	266	(36.9)	189	77		
	More than two	384	(53.3)	228	156	9.59	0.008
	Missing	18	(2.5)				
Working							
	Yes	524	(72.7)	300	224		
	No	188	(26.1)	150	38	30.21	< 0.001
	Missing	9	(1.2)				
Annual personal income							
At diagnosis							
	Less than $20,000	236	(32.7)	138	98		
	$20,000–$39,999	192	(26.6)	111	81		
	More than $40,000	155	(21.5)	87	68	0.22	0.898
	Missing	138	(19.1)				
At time of study							
	Less than $20,000	306	(42.4)	180	126		
	$20,000–$39,999	140	(19.4)	79	61		
	More than $40,000	100	(13.9)	55	45	0.54	0.763
	Missing	175	(24.3)				

*OR* odds ratio

**Table 3:** Patient clinical characteristics (1)

Characteristics		*N*		Mean (SD)
Age at diagnosis				
	Male	308		63.04 (10.2)
	Female	405		54.59 (11.3)
Cancer type				
	Lung	131		
	Breast	201		
	Digestive tract	144		
	Liver/bile duct	47		
	Pancreas	32		
	Urogenital	39		
	Gynecologic	56		
	Head and neck	34		
	Blood	77		
	Others	26		
Multiple cancer diagnosis				
	No	656		
	Yes	59		
	Missing	6

**Table 4:** Patient clinical characteristics (2) and results of univariate analysis

		Total			Request			
Characteristics		*N*	(%)		No	Yes	OR	*p* value
Treatment status								
	Currently receiving	498	(69.1)		328	170		
	Under inspection	192	(26.6)		109	83		
	Completed	19	(2.6)		10	9	5.83	0.054
	Missing	12	(1.7)					
Duration of cancer therapy								
	Less than 6 months	243	(33.7)		149	94		
	6 months–1 year	131	(18.2)		81	50		
	1–2 years	102	(14.1)		67	35		
	2–3 years	57	(7.9)		33	24		
	3–5 years	77	(10.7)		51	26		
	More than 5 years	89	(12.3)		58	31	1.84	0.872
	Missing	22	(3.1)					

*SD* standard deviation, *OR* odds ratio

The relationships between a preference for weekend chemotherapy and sociodemographic and clinical factors were analyzed; results are shown in Tables 2 and 4. Patients who were younger, female, with no spouse/partner, living alone, and employed most commonly indicated a preference for evening and weekend chemotherapy. Multivariate analysis showed that age and employment status were significantly associated with a preference for weekend chemotherapy (Table 5).

**Table 5:** Results of logistic regression analysis of relationships between a desire for weekend outpatient chemotherapy and various factors

	OR	*p* value
Age group	0.388	< 0.001
Gender	1.332	0.123
Marital status	0.735	0.161
Living situation	1.11	0.479
Working	2.173	< 0.001

*OR* odds ratio

## Discussion

To the best of our knowledge, this is the first study to evaluate interest in evening and weekend outpatient chemotherapy. Outpatient chemotherapy services are important for maintenance of patient QoL; however, outpatient treatment requires frequent visits to the hospital, which may be socially burdensome for some patients with cancer. To reduce the social burden on patients with cancer, we explored whether there was an unmet demand for evening or weekend chemotherapy.

In our study, nearly 37% of patients expressed interest in weekend outpatient chemotherapy. Notably, in a study of the employment and economic burdens posed by cancer treatment, two common social costs of cancer, 21% of Japanese patients took early retirement due to cancer [13], while 44% reported a financial burden imposed by cancer treatment [14]. Our data showed that a similar number of patients reported a burden associated with the availability of outpatient anticancer chemotherapy on weekdays alone.

Possible burdens of outpatient chemotherapy for patients with cancer include (i) a direct burden to travel to and from the hospital, such as transportation cost and physical effort, (ii) social burdens required for hospital visits, such as taking time off of work, and so on. Several factors, either alone or in combination, may influence the desire for weekend outpatient chemotherapy. For example, younger patients might have more work and a busier social life [15, 16], which would increase their interest in weekend treatment. Furthermore, older patients might experience difficulty in traveling to the hospital [17] and would therefore also prefer a weekend treatment option. In our study, factors presumably related to the travel burden, such as proximity to the hospital or reliance on a spouse or cohabitant for assistance in traveling to the hospital, were identified in univariate analysis. However, multivariate analysis found that age and employment status, but not direct burden to travel, were significantly associated with a desire for weekend chemotherapy. These results suggested that it was difficult for patients with cancer to allocate sufficient time to visit the hospital, whereas travel itself was a smaller burden.

As a demographic factor, age can be confounded by other factors, such as the higher likelihood of employment among younger patients. However, age remained significant in multivariate analysis, even after employment was eliminated as a potentially confounding factor. Although adolescents and young adults comprise a small percentage of patients with cancer, their medical needs are often unmet [15, 18] and specific types of support may be required [18, 19]. For example, adolescent and young adult patients may require greater effort to visit the farther and more specialized hospital [20, 21]. Younger patients have a larger social role and are thus likely to view outpatient hospital visits as burdensome, which would explain their interest in a weekend chemotherapy option.

Employment is also a major social consideration for patients with cancer [22], and employment itself has been shown to improve their QoL [23, 24]. Since 2012, the Japanese government has promoted opportunities for patients with cancer to continue employment or to be re-employed at their jobs, through the Basic Plan to Promote Cancer Control Program [25]. An equally common social problem of patients with cancer is income [26, 27], which is directly related to employment [28, 29]. Thus, the desire for weekend chemotherapy may have been linked to income. However, our data suggested that employment itself played a larger role than income in the desire for weekend chemotherapy. This might have been due to the importance of employment in Japan. Takahashi et al. found that, among Japanese patients with cancer, the main reasons for leaving a job were “I did not want to be a burden at my workplace,” “I anticipated a lack of energy and physical strength for work,” and “I was not confident that I could balance cancer treatment and work” [13]. These findings were presumed to reflect the greater value placed by Japanese patients with cancer on the impact of their treatment with respect to their employer and colleagues, rather than the loss of income due to lack of employment.

The patients in our survey reported that low effort was needed to visit the hospital. Several reports in other countries found that the effort needed to visit the hospital, such as the distance that had to be traveled, affected the prognosis. In Japan, the burden of visiting the hospital may be less than in other countries because of free access to the hospital and the relative ease of travel to local hospitals [21, 30].

Our study was based on an open-ended survey format with hundreds of respondents across multiple hospitals. However, our questionnaire-based approach had several limitations; therefore, our results may not be generalizable to all patients with cancer. Participants were treated at multiple cancer treatment centers, which may have confounded the results. Moreover, social factors are complex, and this survey did not consider all possible trends. It should also be noted that, despite patient interest in evening and weekend chemotherapy, adjustments to weekly chemotherapy availability may require additional medical staff, materials, and changes in the hospital work environment. While some hospitals are able to offer evening and weekend chemotherapy, this may not be feasible for all hospitals, as it would require major changes in their healthcare systems. However, our survey results may be of interest to institutions considering alternative treatment schedules.

In summary, the interest of patients with cancer in evening and weekend outpatient chemotherapy was influenced by age and employment status. Meeting this medical need may alleviate a social burden. Thus, along with efforts to reduce the physical side effects of chemotherapy, efforts should be expended to reduce the social burden, such as by providing evening and weekend outpatient chemotherapy.

## References

[1] Chen L, Linden HM, Anderson BO, Li CI (2014). Trends in 5-year survival rates among breast cancer patients by hormone receptor status and stage. Breast Cancer Res Treat.

[2] Iversen LH, Green A, Ingeholm P, Østerlind K, Gögenur I (2016). Improved survival of colorectal cancer in Denmark during 2001–2012: the efforts of several national initiatives. Acta Oncol (Madr).

[3] Takano N, Ariyasu R, Koyama J, Sonoda T, Saiki M, Kawashima Y, Oguri T, Hisakane K, Uchibori K, Nishikawa S, Kitazono S, Yanagitani N, Ohyanagi F, Horiike A, Gemma A, Nishio M (2019). Improvement in the survival of patients with stage IV non-small-cell lung cancer: experience in a single institutional 1995–2017. Lung Cancer.

[4] Uramoto H, Kagami S, Iwashige A, Tsukada J (2007). Evaluation of the quality of life between inpatients and outpatients receiving cancer chemotherapy in Japan. Anticancer Res.

[5] Matsuda A, Kobayashi M, Sakakibara Y (2011). Quality of life of lung cancer patients receiving outpatient chemotherapy. Exp Ther Med.

[6] Sultan A, Pati AK, Choudhary V, Parganiha A (2018). Hospitalization-induced exacerbation of the ill effects of chemotherapy on rest-activity rhythm and quality of life of breast cancer patients: a prospective and comparative cross-sectional follow-up study. Chronobiol Int.

[7] Hinz A, Weis J, Faller H, Brähler E, Härter M, Keller M, Schulz H, Wegscheider K, Koch U, Geue K, Götze H, Mehnert A (2018). Quality of life in cancer patients—a comparison of inpatient, outpatient, and rehabilitation settings. Support Care Cancer.

[8] Yamada K, Yoshida T, Zaizen Y, Okayama Y, Naito Y, Yamashita F, Takeoka H, Mizoguchi Y, Yamada K, Azuma K (2011). Clinical practice in management of hydration for lung cancer patients receiving cisplatin-based chemotherapy in Japan: a questionnaire survey. Jpn J Clin Oncol.

[9] Asadi-Lari M, Packham C, Gray D (2003). Unmet health needs in patients with coronary heart disease: implications and potential for improvement in caring services. Health Qual Life Outcomes.

[10] Ricci RP, Vicentini A, D’Onofrio A, Sagone A, Vincenti A, Padeletti L, Morichelli L, Fusco A, Vecchione F, Lo Presti F, Denaro A, Pollastrelli A, Santini M (2013). Impact of in-clinic follow-up visits in patients with implantable cardioverter defibrillators: demographic and socioeconomic analysis of the TARIFF study population. J Interv Card Electrophysiol.

[11] Siegel RL, Miller KD, Jemal A (2017). Cancer statistics, 2017. CA Cancer J Clin.

[12] Japan Center for Cancer Control and Information Services (2018) Projected Cancer Statistics, 2018. 2018:4–5. https://ganjoho.jp/en/public/statistics/short_pred.html Accessed 3 Mar 2020

[13] Takahashi M, Tsuchiya M, Horio Y, Funazaki H, Aogi K, Miyauchi K, Arai Y (2018). Job resignation after cancer diagnosis among working survivors in Japan: timing, reasons and change of information needs over time. Jpn J Clin Oncol.

[14] Irwin B, Kimmick G, Altomare I, Marcom PK, Houck K, Zafar SY, Peppercorn J (2014). Patient experience and attitudes toward addressing the cost of breast cancer care. Oncologist.

[15] Warner EL, Kent EE, Trevino KM, Parsons HM, Zebrack BJ, Kirchhoff AC (2016). Social well-being among adolescents and young adults with cancer: a systematic review. Cancer.

[16] Ferrari A, Barr RD (2017) International evolution in AYA oncology: current status and future expectations. Pediatr blood Cancer 64:. 10.1002/pbc.26528 10.1002/pbc.26528 28370975

[17] Krishnasamy C, Unsworth CA, Howie L (2013). Exploring the mobility preferences and perceived difficulties in using transport and driving with a sample of healthy and outpatient older adults in Singapore. Aust Occup Ther J.

[18] Kaal SEJ, Husson O, van Duivenboden S, Jansen R, Manten-Horst E, Servaes P, Prins JB, van den Berg SW, van der Graaf WTA (2017). Empowerment in adolescents and young adults with cancer: relationship with health-related quality of life. Cancer.

[19] Bradford N, Walker R, Cashion C, Henney R, Yates P (2020). Do specialist youth cancer services meet the physical, psychological and social needs of adolescents and young adults? A cross sectional study. Eur J Oncol Nurs.

[20] Alvarez E, Keegan T, Johnston EE, Haile R, Sanders L, Saynina O, Chamberlain LJ (2017). Adolescent and young adult oncology patients: disparities in access to specialized cancer centers. Cancer.

[21] Warner EL, Fowler B, Pannier ST, Salmon SK, Fair D, Spraker-Perlman H, Yancey J, Randall RL, Kirchhoff AC (2018). Patient navigation preferences for adolescent and young adult cancer services by distance to treatment location. J Adolesc Young Adult Oncol.

[22] Kotani H, Kataoka A, Sugino K, Iwase M, Onishi S, Adachi Y, Gondo N, Yoshimura A, Hattori M, Sawaki M, Iwata H (2018). The investigation study using a questionnaire about the employment of Japanese breast cancer patients. Jpn J Clin Oncol.

[23] Rasmussen DM, Elverdam B (2008). The meaning of work and working life after cancer: an interview study. Psychooncology.

[24] Eriksson L, Öster I, Lindberg M (2016). The meaning of occupation for patients in palliative care when in hospital. Palliat Support Care.

[25] The Ministry of Health, Labour and Welfare. The second basic plan to promote cancer control programs (in Japanese) https://www.mhlw.go.jp/stf/seisakunitsuite/bunya/0000183313.html Accessed 3 Mar 2020

[26] Ell K, Xie B, Wells A, Nedjat-Haiem F, Lee PJ, Vourlekis B (2008). Economic stress among low-income women with cancer: effects on quality of life. Cancer.

[27] Myong J, Kim H (2012). Impacts of household income and economic recession on participation in colorectal cancer screening in Korea. Asian Pacific J Cancer Prev.

[28] Guy GP, Ekwueme DU, Yabroff KR (2013). Economic burden of cancer survivorship among adults in the United States. J Clin Oncol.

[29] Kochovska S, Luckett T, Agar M, Phillips JL (2018). Impacts on employment, finances, and lifestyle for working age people facing an expected premature death: a systematic review. Palliat Support Care.

[30] Thomas AA, Gallagher P, O’Céilleachair A, Pearce A, Sharp L, Molcho M (2014). Distance from treating hospital and colorectal cancer survivors’ quality of life: a gendered analysis. Support Care Cancer.

